# Adaptation and Validation of the Chinese Version of the Digital Self-Efficacy Scale in Chinese First-Year College Students: A Bifactor-ESEM Approach

**DOI:** 10.3390/bs16060975

**Published:** 2026-06-12

**Authors:** Jingyi Hu, Qian Gu, Chong Yang, Chuanhua Gu

**Affiliations:** 1 Key Laboratory of Adolescent Cyberpsychology and Behavior (CCNU), Ministry of Education, Wuhan 430079, China; hujingyi@mails.ccnu.edu.cn (J.H.); guqian2023@163.com (Q.G.); 2 Key Laboratory of Human Development and Mental Health of Hubei Province, School of Psychology, Central China Normal University, Wuhan 430079, China; 3 Student Affairs Office, Guangdong University of Finance and Economics, Guangzhou 528100, China; chongyang@gdufe.edu.cn

**Keywords:** digital self-efficacy, scale adaptation, first-year college students, Bifactor-ESEM

## Abstract

As digital technology becomes increasingly embedded in higher education, assessing students’ confidence in digital tasks is essential for understanding their adaptation to digital learning environments. This study adapted the Digital Self-Efficacy Scale (DSES) into Chinese and evaluated its psychometric properties among 1502 first-year college students in China. Participants were randomly split into two subsamples for item analysis and exploratory factor analysis, and structural validation respectively. All 25 items demonstrated satisfactory discrimination and homogeneity. Although parallel analysis indicated a four-factor exploratory solution, seven competing models were compared in the confirmatory stage. The Bifactor-ESEM model yielded the best combination of statistical fit and substantive interpretability, suggesting that the Chinese DSES primarily captures an overarching digital self-efficacy dimension, with domain-specific factors retaining limited reliable variance beyond the general factor. Total scores were positively associated with digital maturity (r = 0.642, *p* < 0.001); however, external validity is limited given that both measures were self-reported and concurrently collected. Gender measurement invariance analyses supported configural, metric, and scalar invariance. Overall, the Chinese DSES demonstrates promising preliminary psychometric properties. The total score is recommended as the primary interpretive unit, with subscale scores used as supplementary descriptive information only.

## 1. Introduction

### 1.1. Digital Self-Efficacy

Digital technology now permeates higher education, shaping how courses are taught, how students learn, and how performance is assessed (Zhao et al., 2021). When entering university, students must adapt not only to new learning materials but also to digitally mediated modes of instruction, collaboration, and assessment. They are also expected to remain engaged while navigating online platforms, collaborating with peers, searching for information, and producing digital content. During this transition, objective computer skills alone do not determine if students will tackle digital tasks or push through technical issues. Rather, students’ beliefs about their own digital capabilities may play a central role in whether they approach, persist in, or avoid digital tasks (Peiffer et al., 2020).

This belief in one’s ability to handle digital tasks is referred to as digital self-efficacy. Bandura’s (1977) social cognitive theory posits that self-efficacy mediates and regulates the relationship between individual actual abilities and behavioral performance. Recent empirical studies have further shown that digital self-efficacy is not only a key predictor of ICT skills (Hatlevik, 2017), but also promotes or hinders the development of digital interaction abilities by influencing individual acceptance of new technologies (Ertmer et al., 1994; Hatlevik et al., 2018). For example, internet self-efficacy can predict individual online learning performance (Chang et al., 2014; Joo et al., 2000). In turn, frequent use of digital systems reinforces digital self-efficacy, forming a reciprocal feedback loop (Eastin & LaRose, 2000).

Importantly, digital self-efficacy is more than a reflection of perceived digital ability (Marakas et al., 1998); it can also shape objective digital performance by guiding motivational processes and behavioral choices in human-technology interactions. In other words, digital self-efficacy extends beyond operational know-how; it captures a person’s confidence in mobilizing digital skills to solve problems and navigate challenges in online environments (Ulfert-Blank & Schmidt, 2022).

To understand how first-year students adapt to digital learning environments, it is not enough to assess their mastery of specific software; researchers must also gauge whether students believe they can navigate these environments effectively (Ulfert-Blank & Schmidt, 2022). A psychometrically sound measure of digital self-efficacy is therefore essential—both for advancing basic research and for informing the design of digital education and student support services in universities.

### 1.2. The Digital Self-Efficacy Scale

There are several frameworks for assessing digital competence, such as the European Union’s Digital Competence Framework (DigComp), which divides digital competence into five objective domains: information and data literacy, communication and collaboration, digital content creation, safety, and problem-solving (Carretero et al., 2017; Vuorikari et al., 2022; Vuorikari et al., 2016). However, DigComp is fundamentally an objective competence framework and should not be conflated with individuals’ subjective beliefs about their own abilities. Psychological research has repeatedly shown that abilities and ability beliefs should be distinguished, as both independently influence individual learning, motivation, and performance (Hughes et al., 2011; Marsh et al., 2017; Pajares & Schunk, 2002).

Ulfert-Blank and Schmidt (2022) developed the Digital Self-Efficacy Scale (DSES) based on the DigComp framework, recasting the objective competence descriptors as self-efficacy items across these five domains. The 25-item scale is grounded in a clear theoretical framework with broad content coverage, making it one of the few digital self-efficacy instruments derived directly from DigComp. Although the scale has been adapted into Spanish (Paredes-Aguirre et al., 2024), its latent structure has not yet been psychometrically examined among Chinese first-year college students. Given differences in educational contexts, digital learning practices, and student developmental stages, it remains an open empirical question whether the original five-domain structure is clearly distinguishable or reorganized in this population.

### 1.3. Expected Latent Structure in the Chinese Context

In cross-cultural measurement, adapting a scale goes beyond literal translation; it requires re-examining whether the target construct retains the same empirical organization in the new population. Among Chinese first-year college students, the structure of digital self-efficacy may be influenced by educational, technological, and developmental factors, and thus may not perfectly reproduce the five-domain structure assumed by the original scale.

First, Chinese higher education increasingly relies on integrated digital learning platforms. After the COVID-19 pandemic, universities have widely adopted platforms such as Rain Classroom, Chaoxing Learning, and Blue Ink Cloud Class to support hybrid teaching (Y. Hu et al., 2024; Jia, 2024). On these platforms, students often search for information, collaborate on documents, verify identities, submit assignments, and receive feedback within a single workflow. As a result, theoretically distinct domains such as communication, safety, and problem-solving may become less clearly differentiated in students’ subjective experience.

Second, first-year college students are still developing autonomous learning strategies and differentiated self-appraisals. When confronting complex technical tasks, individuals may initially rely on a global sense of control before forming more domain-specific judgments (Xu et al., 2025). Therefore, although the Chinese DSES may retain the five DigComp domains as a content framework, its empirical structure may also contain a strong general factor reflecting broad digital self-efficacy. The present study therefore examined whether the scale is better represented by five distinguishable domain-specific factors, an overarching digital self-efficacy dimension, or a structure combining both general and domain-specific components.

### 1.4. Model Comparison Strategy

Self-efficacy measurement is typically divided into two categories: general scales and domain or task-specific scales (Gecas, 1989). General self-efficacy reflects a broad, trait-like belief in one’s ability to cope effectively across diverse situations (G. Chen et al., 2001), whereas domain- or task-specific self-efficacy captures confidence in performing particular tasks within defined contexts (Hatlevik et al., 2018).

Researchers widely use confirmatory factor analysis (CFA) to model multidimensional variables. However, CFA poses two problems for closely related constructs like digital self-efficacy. First, it relies on the strict and often unrealistic independent cluster model, assuming each item loads on only one factor. Second, it fails to evaluate the common and unique effects of these dimensions simultaneously. When CFA yields a poor fit, more flexible modeling approaches are warranted (Gu & Wen, 2025).

Exploratory structural equation modeling (ESEM) allows small cross-loadings under theoretical constraints, better adapting to the reality of interconnected digital tasks (Marsh et al., 2014; Morin et al., 2016). However, if researchers further suspect that the common variance between items comes not only from dimensional correlation but may also stem from an overarching general factor, ESEM alone still cannot separate the true specific variance (Reise, 2012; Rodriguez et al., 2016).

The bifactor model provides a more suitable analytical path. This model simultaneously allocates each item’s variance to a general factor and several specific factors, thereby distinguishing “common variance shared by all items” from “residual variance unique to a specific content domain”. The Bifactor-ESEM framework proposed by Morin et al. (2016) combines ESEM with the bifactor model and recommends a sequential model comparison strategy: first compare CFA and ESEM to determine if cross-loadings are non-negligible; if ESEM outperforms CFA, then compare ESEM and Bifactor-ESEM to determine if there is a strong general factor (Morin et al., 2016). The primary aim of this sequential strategy is not to maximize statistical fit per se, but to answer a substantive measurement question: Is variation in Chinese DSES responses driven primarily by a broad digital self-efficacy dimension, by five distinguishable domain-specific factors, or by both sources of variance? The answer directly determines whether future research should use the total scale score or subscale scores.

Given concerns that bifactor models may sometimes improve fit through statistical flexibility rather than substantive distinctiveness, the present study interpreted the Bifactor-ESEM solution not only by global fit indices but also by bifactor reliability indices, explained common variance, and theoretical interpretability. This caution is consistent with previous psychometric discussions warning that bifactor models should not be accepted solely on the basis of superior fit indices.

### 1.5. The Present Study

The present study aimed to adapt the Digital Self-Efficacy Scale (DSES) into Chinese and to examine its psychometric properties among Chinese first-year college students. Specifically, the study had four objectives. First, following international guidelines for scale adaptation (Brislin, 1970; International Test Commission, 2017), we translated and culturally adapted the original scale to develop a Chinese version of the DSES suitable for first-year college students in China. Second, using independent subsamples, we conducted item analysis, exploratory factor analysis, and competing model comparisons to systematically examine the latent structure of the Chinese version of the DSES. This comparison encompassed both data-driven models suggested by the exploratory findings and theory-driven models derived from the original DigComp-based framework, including four-factor, five-factor, second-order, bifactor CFA, and Bifactor-ESEM specifications. Third, based on the best-fitting and most theoretically interpretable model, we further evaluated the reliability of the scale, the basis for structural interpretation, and its preliminary association with digital maturity, thereby informing decisions about whether to interpret the total score, the subscale scores, or both. Fourth, we examined gender measurement invariance using a sequential multi-group modeling approach—testing configural, metric, and scalar invariance across male and female students—to establish whether the Chinese DSES produces comparable measurements across gender groups. Given the integrated nature of digital learning platforms in Chinese higher education and the developmental characteristics of first-year students, we examined whether the Chinese DSES would retain the five-domain content framework of DigComp, show a strong general factor, or be best represented by a structure combining both general and domain-specific components. It should be noted that the cultural factors emphasized in the following arguments represent plausible contextual interpretations; the present design cannot empirically isolate whether any observed structural pattern is uniquely attributable to the Chinese educational context or reflects a broader psychometric characteristic of the DSES itself.

## 2. Materials and Methods

### 2.1. Translation and Adaptation of the Original Scale

The scale adapted in the present study was the Digital Self-Efficacy Scale (DSES) developed by Ulfert-Blank and Schmidt (2022). The original scale consists of 25 items based on the DigComp framework and corresponds to five theoretical domains: information and data literacy, communication and collaboration, digital content creation, safety, and problem-solving. All items are rated on a 6-point scale (Ulfert-Blank & Schmidt, 2022). Before beginning the formal adaptation, we obtained permission from the original authors to use and adapt the scale.

The development of the Chinese version of the DSES followed Brislin’s (1970) translation–back-translation procedure and the International Test Commission guidelines for test adaptation (International Test Commission, 2017), and was supplemented by expert review and cognitive interviewing. First, two doctoral students in psychology independently translated the English version into Chinese. Second, the research team, consisting of experts in educational psychology, psychometrics, and digital learning, reviewed the two translations item by item. They reconciled differences in wording and tone based on criteria of semantic equivalence, cultural relevance, and clarity, while staying as close to the original meaning as possible. This process yielded the initial Chinese draft. Third, a bilingual expert with no prior exposure to the original scale independently back-translated the Chinese draft into English. Fourth, we performed informal brief open-ended interviews with ten first-year college students, who commented on potentially ambiguous wording. These procedures provided preliminary evidence of linguistic and conceptual appropriateness, although more extensive cognitive interviewing should be conducted in future research. Fifth, the revised back-translated version was compared item by item against the original English version to verify conceptual equivalence.

Following these procedures, the final item pool of the Chinese version of the DSES retained all 25 original items, with minor wording adjustments made to improve clarity. The content framework and response format were kept consistent with those of the original scale. Thus, the adaptation process went beyond literal translation. It involved semantic calibration, cultural adjustment, expert content review, and student-informed revision, providing preliminary support for the linguistic and conceptual appropriateness of the Chinese version in the local context.

### 2.2. Participants and Procedure

We recruited a convenience sample of first-year students from two universities in Guangzhou, Guangdong Province, China: one comprehensive university and one vocational college. The students’ academic disciplines covered the humanities, sciences, and engineering. For the purposes of initial psychometric validation of the Chinese DSES, this sample provides sufficient structural diversity across institution type, academic discipline, and socioeconomic background. Convenience sampling is consistent with standard practice in scale development and validation research, in which the primary goal is to evaluate the psychometric properties of the instrument rather than to estimate population parameters (Worthington & Whittaker, 2006). Questionnaires were administered through an online survey platform, and participation was voluntary. A total of 1550 questionnaires were collected. After data screening, 48 responses were excluded: 10 because of incomplete data, 20 because of duplicate submissions or duplicate IP addresses, and 18 because of unrealistically short completion time. The final analytic sample consisted of 1502 valid responses.

Using SPSS 29.0, the 1502 valid responses were randomly divided into two independent subsamples. Subsample 1 consisted of 750 participants and was used for item analysis and exploratory factor analysis; it included 337 males (44.9%) and 413 females (55.1%), with a mean age of 18.49 years (SD = 0.68). Subsample 2 consisted of 752 participants and was used for structural validation and competing model comparison; it included 414 males (55.1%) and 338 females (44.9%), with a mean age of 18.84 years (SD = 1.45). The survey included the Chinese version of the Digital Self-Efficacy Scale and the Digital Maturity Inventory. After providing informed consent, all participants completed the questionnaires anonymously. The study was approved by the ethics committee of the authors’ institution (approval number: IRB-202501011b).

Although the split was random, the two subsamples differed slightly in age composition. This difference should be considered when interpreting cross-sample comparisons, although the primary analyses relied on independent subsamples for distinct exploratory and confirmatory purposes.

### 2.3. Measures

#### 2.3.1. Chinese Version of the Digital Self-Efficacy Scale (DSES)

The Chinese version of the Digital Self-Efficacy Scale (DSES) was developed in the present study based on the original instrument. It contains 25 items corresponding to the five theoretical domains of the DigComp framework: information and data literacy, communication and collaboration, digital content creation, safety, and problem-solving. The scale uses a 6-point response format (Ulfert-Blank & Schmidt, 2022). The Chinese Version of the DSES Items is provided in Appendix B.

Although the scale retains the original five-domain content framework, we did not assume a priori that a simple five-factor structure would emerge among Chinese first-year college students. Its latent structure was therefore examined systematically through item analysis, exploratory factor analysis, and competing model comparisons.

#### 2.3.2. Digital Maturity Inventory (DIMI)

To evaluate the external validity of the Chinese DSES, we included the Digital Maturity Inventory (DIMI) as a criterion measure (Laaber et al., 2023). The DIMI consists of 32 items rated on a 5-point scale and covers three broad areas: autonomous use of digital technology, coping with digital challenges, and social interaction and participation in digital environments.

As no published Chinese version of the DIMI was available, we developed a Chinese trial version following the same translation–back-translation and expert review procedures as used for the DSES.

In the present sample (N = 1502), the Chinese version of the DIMI showed satisfactory internal consistency and structural validity: Cronbach’s α = 0.890; confirmatory factor analysis yielded χ^2^/*df* = 2.96, CFI = 0.947, TLI = 0.937, RMSEA = 0.051, and SRMR = 0.055. All fit indices reached acceptable levels, supporting the appropriateness of the DIMI for use in this sample.

We used the correlation between the total score of the DIMI and the total score of the Chinese version of the DSES as initial evidence of convergent and external validity. Because both instruments rely on self-report and assess related but non-identical aspects of digital functioning (the DSES measures digital self-efficacy, whereas the DIMI measures digital maturity), this analysis was designed to provide preliminary external support rather than a stringent criterion-validity test.

### 2.4. Data Analytic Strategy

We used a split-sample design that combined exploratory and confirmatory analyses to examine the latent structure of the Chinese DSES. This strategy allowed us to use one subsample to obtain an initial empirical understanding of the item structure and another independent subsample to test competing structural models. The overall analytic procedure proceeded in four sequential stages.

Stage 1: Item Analysis and Exploratory Factor Analysis (Subsample 1). Item analysis focused on descriptive statistics, item discrimination, and item homogeneity. Item discrimination was assessed using the extreme-group method, in which respondents in the upper and lower 27% of the total-score distribution were compared, and independent-samples *t* tests were used to calculate the critical ratio (CR) for each item. Item homogeneity was evaluated using corrected item–total correlations (CITCs) and Cronbach’s α if the item was deleted. Exploratory factor analysis (EFA) was then conducted to obtain an initial empirical picture of the scale’s latent structure, with parallel analysis (PA) used as a statistical guide for factor retention.

Parallel analysis was treated as an important exploratory indicator but not as a mechanical decision rule for the confirmatory stage. In cross-cultural scale adaptation, decisions about factor structure should consider not only empirical extraction results but also theoretical interpretability, content coverage, and cross-sample replicability (DeVellis & Thorpe, 2021). Therefore, the confirmatory stage was designed to evaluate both the data-driven factor solution suggested by PA and the theory-driven five-domain structure derived from the original DigComp-based DSES framework, rather than mechanically carrying forward only the exploratory factor count.

Stage 2: Competing Model Comparisons and Structural Validation (Subsample 2). Structural validation and competing model comparisons were conducted in Subsample 2 using Mplus 8.3. Robust maximum likelihood estimation (MLR) was used as the primary estimator for all structural models. Because the DSES items were rated on a 6-point Likert-type scale and the sample size was large, the items were treated as approximately continuous variables. MLR was selected because it provides robust standard errors and fit indices under non-normality and allows direct comparison across CFA, ESEM, bifactor CFA, and Bifactor-ESEM models.

Following the sequential model comparison strategy proposed by Morin et al. (2016), the confirmatory stage proceeded in two primary steps. In Step 1, the five-factor oblique CFA model was compared with the five-factor ESEM model to determine whether allowing theoretically plausible cross-loadings improved the representation of the Chinese DSES. In Step 2, the five-factor ESEM model was compared with the Bifactor-ESEM model to determine whether item responses were better explained by both an overarching general digital self-efficacy factor and residual domain-specific factors (Morin et al., 2016). To provide a more comprehensive evaluation of alternative latent structures, three additional models were also included: a four-factor CFA model and a four-factor ESEM model based on the empirical pattern suggested by the exploratory analysis in Subsample 1, and a second-order CFA model based on the original five-domain framework. The inclusion of four-factor confirmatory models directly followed up on the exploratory findings, allowing us to test whether the data-driven factor solution could be replicated and whether it was empirically superior to the theory-driven alternatives. Thus, seven models were compared in total: a four-factor CFA model, a four-factor ESEM model, a five-factor oblique CFA model, a second-order CFA model, a five-factor ESEM model, a bifactor CFA model, and a Bifactor-ESEM model.

Both the ESEM and Bifactor-ESEM models used target rotation. In the Bifactor-ESEM model, all items were specified to load on a general digital self-efficacy factor and on their target-specific factors, while non-target cross-loadings were freely estimated but targeted to be close to zero. The general factor and the specific factors were specified as orthogonal, consistent with the bifactor modeling framework.

Model fit was evaluated in accordance with the recommendations of L. Hu and Bentler (1999) and Marsh et al. (2014). CFI and TLI values above 0.90 were considered acceptable and values above 0.95 were considered good; RMSEA and SRMR values below 0.08 were considered acceptable and values below 0.05 were considered good (L. Hu & Bentler, 1999; Marsh et al., 2014). Because chi-square values are highly sensitive to large sample sizes, model evaluation relied primarily on approximate fit indices, theoretical interpretability, and the substantive meaning of factor loadings rather than on chi-square alone. Information criteria (AIC and BIC) were also reported to support comparisons among non-nested models.

Stage 3: Sensitivity Analysis Using WLSMV (Subsample 2). We acknowledge that ordinal estimators such as WLSMV are also appropriate for ordered categorical data. Although MLR was adopted as the primary estimator given the six response categories, large sample size, and the need for comparability across the full set of complex models, we conducted a sensitivity analysis in Subsample 2 using the WLSMV estimator to examine whether the primary model-comparison conclusion was robust when the six-point Likert items were treated as ordered categorical indicators. Rather than repeating the full seven-model sequence, the WLSMV sensitivity analysis focused on the two theoretically central models that anchored our comparison: the five-factor oblique CFA model and the Bifactor-ESEM model. This targeted comparison was designed to evaluate estimator robustness rather than to replicate the complete model-selection procedure.

Stage 4: Bifactor Reliability, External Validity, and Gender Measurement Invariance. After identifying the best-fitting and most theoretically interpretable model, we computed bifactor reliability and dimensionality indices to determine whether the Chinese DSES should be interpreted primarily at the total-score level, subscale level, or both. These indices included omega total (ω), omega hierarchical (ω_H_), omega hierarchical subscale (ω_HS_), explained common variance (ECV), and percentage of uncontaminated correlations (PUC). To provide preliminary convergent and external validity evidence, Pearson product–moment correlation analysis was conducted to examine the association between the total score of the Chinese DSES and the total score of digital maturity. Because both measures were self-report instruments assessing related but non-identical aspects of digital functioning, this analysis was interpreted as preliminary external validity evidence rather than as a stringent criterion-validity test (Rodriguez et al., 2016).

Finally, gender measurement invariance of the optimal model was examined using multi-group modeling. Configural, metric, and scalar invariance models were tested sequentially across male and female students. Because chi-square difference tests are sensitive to large sample sizes, invariance decisions were based primarily on changes in approximate fit indices, including ΔCFI, ΔRMSEA, and ΔSRMR. Following F. F. Chen’s (2007) recommended criteria, changes in ΔCFI ≤ 0.010, ΔRMSEA ≤ 0.015, and ΔSRMR ≤ 0.030 for metric invariance, or ΔSRMR ≤ 0.010 for scalar invariance, were taken as evidence supporting measurement invariance (F. F. Chen, 2007).

## 3. Results

### 3.1. Descriptive Statistics and Item Analysis

As shown in Table 1, the items of the Chinese DSES exhibited satisfactory psychometric properties. Item means ranged from 3.81 to 4.55 (SDs = 0.91–1.19), with no evidence of floor or ceiling effects. Skewness values ranged from −0.66 to −0.20 and kurtosis from −0.21 to 1.46, suggesting approximate normality across items.

To evaluate item discrimination, we compared the top and bottom 27% of respondents using independent-samples *t* tests. Critical ratios (CRs) for the 25 items ranged from 17.15 to 29.08 (*p* < 0.001), confirming that every item adequately distinguished between high- and low-scoring respondents.

Corrected item-total correlations (CITCs) ranged from 0.64 to 0.83, all well above the conventional 0.30 threshold. Item-deletion analyses further showed that Cronbach’s α did not increase upon deletion of any single item, indicating that each item contributed positively to overall internal consistency.

In sum, all 25 items showed adequate discrimination, homogeneity, and internal consistency and were retained for subsequent structural analyses.

### 3.2. Exploratory Factor Analysis

Before running the EFA, we assessed whether the data were suitable for factor analysis. The results showed that the KMO value was 0.973, and Bartlett’s test of sphericity was significant, χ^2^(300) = 18,606.054, *p* < 0.001, indicating that the sample was suitable for factor analysis.

Common factor-retention rules—such as the eigenvalue-greater-than-one criterion or scree-plot inspection—are partly subjective and sensitive to both sample size and the number of variables. For instance, the eigenvalue-greater-than-one criterion tends to overextract factors, particularly when many items are involved. Parallel analysis (PA) compares the actual eigenvalues with the 95th percentile eigenvalues generated from random data matrices of the same size and retains a factor only when its actual eigenvalue exceeds the random threshold (Braeken & Van Assen, 2017; Costello & Osborne, 2005; Hayton et al., 2004; Zwick & Velicer, 1986). Because parallel analysis does not rely on subjective cutoffs, it is now widely recommended as a factor-retention criterion. Accordingly, parallel analysis was used in the present study.

As shown in Table 2, the actual eigenvalues of the first four factors (14.97, 1.51, 0.57, and 0.36) all exceeded their corresponding simulated thresholds (0.40, 0.31, 0.27, and 0.23), whereas the fifth actual eigenvalue (0.15) was lower than the corresponding random threshold (0.20). Thus, the results of parallel analysis supported the extraction of four factors. However, DigComp defines content domains rather than prescribing a particular latent structure; there is no guarantee that its five domains will emerge as statistically distinct factors in every sample. We therefore retained the five-factor solution as a theoretically motivated candidate but did not presume it would be optimal for the present sample.

Based on parallel analysis, the four-factor exploratory model demonstrated good overall fit (χ^2^(206) = 557.646, *p* < 0.001, RMSEA = 0.048, CFI = 0.963, TLI = 0.946, SRMR = 0.019). However, the exploratory loading matrix (see Appendix A) revealed that the four-factor solution was difficult to interpret theoretically. Specifically, items from information and data literacy (ISE) and communication and collaboration (CSE) predominantly loaded on the same primary factor, blurring the distinction between these core theoretical domains. In addition, several items showed notable cross-loadings and theoretical misalignment. For example, DSE4 had moderate loadings on both Factor 2 and Factor 4 (0.388 and 0.438), and CSE7 showed similar loadings on Factor 1, Factor 2, and Factor 3 (0.327, 0.338, and 0.326). In other words, although the four-factor model fit the data adequately in a statistical sense, it did not yield a factor pattern that could be mapped onto recognizable content domains in a clear way.

Two considerations guided the decision to carry the five-domain framework forward into the confirmatory stage while also formally testing the exploratory four-factor alternative. First, DigComp defines content domains rather than prescribing a fixed latent structure; although the five-domain distinction remains the most widely used content framework for organizing digital competence and digital self-efficacy items (Carretero et al., 2017; Ulfert-Blank & Schmidt, 2022), these domains may not always emerge as fully distinct statistical factors. Second, the purpose of the confirmatory stage was not simply to replicate the exploratory factor count, but to compare whether the data-driven four-factor solution, the original five-domain structure, a higher-order structure, or a bifactor structure provided the most interpretable representation of the Chinese DSES. The four-factor exploratory result was therefore treated as an informative empirical finding and was formally examined in Subsample 2 alongside theory-driven five-domain and bifactor alternatives.

### 3.3. Competing Model Comparison and Structural Validation

To validate the dimensional structure of the Chinese DSES, we employed a cross-validation procedure using Subsample 2 (n = 752). As noted previously, parallel analysis in Subsample 1 suggested extracting four factors, and the exploratory loading matrix indicated a data-driven tendency for items from information and data literacy (ISE) and communication and collaboration (CSE) to load on a common factor. However, the four-factor solution was not theoretically straightforward, and several items showed diffuse cross-loadings.

To evaluate both the data-driven exploratory structure and the original DigComp-based theoretical framework, seven competing models were specified and compared in Subsample 2: a four-factor CFA model, a four-factor ESEM model, a five-factor oblique CFA model, a second-order CFA model, a five-factor ESEM model, a bifactor CFA model, and a Bifactor-ESEM model. The four-factor CFA and ESEM models were included to directly test whether the empirically suggested factor solution could be replicated in an independent confirmatory sample. The five-factor and hierarchical models were included to test whether the original five-domain content framework, a higher-order structure, or a bifactor representation provided a more defensible account of the data. The fit indices for the seven competing models are summarized in Table 3.

Step 1: Comparison between CFA and ESEM. In the first step, we assessed whether cross-loadings were substantively meaningful. The five-factor oblique CFA model served as the baseline; after allowing small non-target loadings, the five-factor ESEM model improved fit relative to CFA: CFI increased from 0.943 to 0.969, RMSEA decreased from 0.050 to 0.044, and SRMR decreased from 0.044 to 0.016. The marked drop in SRMR, in particular, suggests that cross-loadings captured residual covariation that the CFA model had left unmodeled.

Further insight comes from comparing the factor correlation matrices of the two models (see Table 4). In the CFA model, the average inter-factor correlation was as high as 0.82, with several factor pairs approaching 0.87–0.89. In the ESEM model, the average inter-factor correlation dropped to 0.52. This substantial reduction indicates that much of the apparent overlap among factors under CFA was an artifact of constraining cross-loadings to zero. These findings confirm that imposing strict independent-cluster constraints inflates inter-factor correlations and obscures the empirical dimensionality of the scale. ESEM thus provides a more realistic representation of the Chinese DSES, whose content domains are conceptually interrelated.

Regarding the four-factor models, the strict four-factor CFA model showed weaker fit than the five-factor oblique CFA model, suggesting that forcing the merger of ISE and CSE under an independent-cluster framework did not improve the representation of the data. The four-factor ESEM model showed acceptable-to-good fit, indicating that the exploratory four-factor pattern had some empirical support when cross-loadings were allowed. Nevertheless, the five-factor ESEM model showed better fit than the four-factor ESEM model across all indices, suggesting that preserving the five theoretical content domains—rather than collapsing ISE and CSE—yielded a more adequate representation even in the more flexible ESEM framework. These results indicate that the exploratory four-factor pattern did not replicate as a superior confirmatory solution; the five-domain framework better reflected the empirical structure of the data in Subsample 2.

Step 2: Comparison between ESEM and Bifactor-ESEM. Having established that ESEM outperformed CFA, and that the five-factor solution outperformed the four-factor solution, we next examined whether the data were better represented by an additional general factor. The results showed that, after adding an orthogonal general factor to the ESEM model, the Bifactor-ESEM model improved further and provided the best fit among all seven competing models (CFI = 0.985, TLI = 0.973, RMSEA = 0.032, SRMR = 0.012; see Table 3).

The second-order CFA model did not improve fit relative to the five-factor oblique CFA model (ΔCFI = −0.017, ΔRMSEA = 0.006), suggesting that a traditional higher-order representation was insufficient to capture the structure of the data. More importantly, the second-order CFA model fit substantially worse than the Bifactor-ESEM model across all reported indices. This contrast indicates that the general factor was not merely a higher-order abstraction operating through five separable domains; rather, it represented a pervasive source of item-level variance that was more directly captured by the bifactor specification.

The simplified loading summary in Table 5 presents the standardized loadings on the general factor and the theoretically assigned target-specific factors. The full ESEM and Bifactor-ESEM loading matrices are provided in Appendix A. Three aspects of the loading pattern merit attention. First, the overarching dimension showed consistently high loadings across all 25 items, ranging from 0.62 to 0.83, with a mean of approximately 0.76, indicating that item responses were strongly associated with a broad digital self-efficacy dimension across domains. Second, after the general factor was introduced, most non-target cross-loadings in the Bifactor-ESEM solution were small, suggesting that the inter-domain overlap observed in the ESEM solution was largely absorbed by this overarching dimension. Third, after controlling for the general factor, several target-specific factor loadings remained meaningful, but they were generally weaker and less consistent than the general-factor loadings. These results suggest that the five DigComp domains contributed residual content-specific information, but primarily as secondary differentiations beneath a broad digital self-efficacy dimension.

Taken together, the model comparisons support a clear interpretive conclusion: reducing the scale to a strict four-factor structure may overlook theoretically meaningful content distinctions, whereas a traditional five-factor oblique model may overstate the independence of the five domains. Compared with the second-order CFA, the Bifactor-ESEM model better captured the pervasive item-level influence of a general factor. The Bifactor-ESEM model therefore provided both the best statistical fit and the most interpretable account of the Chinese DSES structure: a dominant general digital self-efficacy dimension supplemented by residual domain-specific components.

### 3.4. Robustness, Reliability, and Structural Interpretation of the Bifactor-ESEM Solution

Given that the Bifactor-ESEM model was identified as the most defensible solution in the MLR-based model comparison, a sensitivity analysis was conducted in Sample 2 using the WLSMV estimator to examine whether this conclusion was robust when the six-point Likert items were treated as ordered categorical indicators. The WLSMV-estimated Bifactor-ESEM model showed acceptable fit by RMSEA and excellent fit by CFI, TLI, and SRMR, χ^2^(165) = 838.373, RMSEA = 0.074, CFI = 0.989, TLI = 0.980, SRMR = 0.010. In contrast, the WLSMV-estimated five-factor CFA model showed poorer fit, particularly in terms of RMSEA, χ^2^(265) = 3437.175, RMSEA = 0.126, CFI = 0.947, TLI = 0.940, SRMR = 0.034. These findings were consistent with the MLR-based analyses and indicated that the superiority of the Bifactor-ESEM solution was not driven by the choice of estimator. Therefore, the Bifactor-ESEM solution was retained for subsequent reliability and structural interpretation.

A key advantage of Bifactor-ESEM over traditional CFA is that it helps determine whether scale scores should be interpreted primarily at the total-score level, the subscale level, or both.

Based on the Bifactor-ESEM model, we further calculated a series of bifactor indices for the total score and the specific factors (see Table 6). The results showed that the overall reliability of the total scale was very high (ω = 0.983), and the hierarchical omega of the general factor was also high (ω_H_ = 0.946), indicating that most of the reliable variance in the total score can be attributed to the general digital self-efficacy construct. In addition, the explained common variance of the general factor was ECV = 0.797, indicating that the vast majority of common variance was accounted for by the general factor. Together, these results indicate that the total score of the Chinese DSES primarily reflects a general digital self-efficacy factor that spans all items and domains.

We also report the conventional percentage of uncontaminated correlations (PUC = 0.810) as a supplementary descriptive index. This value was computed using the theoretical item-domain assignment—that is, by counting the proportion of inter-item correlations that involve items from different domains. PUC was originally proposed for bifactor models with independent-cluster item assignments (Rodriguez et al., 2016), and its interpretation becomes less straightforward in Bifactor-ESEM models where every item is allowed to cross-load on non-target factors. We therefore do not treat PUC as a decisive criterion on its own. Nevertheless, considered jointly with the high ω_H_ and ECV values, the observed PUC exceeds the 0.70 threshold below which Rodriguez et al. (2016) cautioned that bias in bifactor-based indices may become substantial. On balance, the convergence of ω_H_ = 0.946, ECV = 0.797, and PUC = 0.810 supports prioritizing the total score as the primary unit of interpretation.

Further, the ω_HS_ values of the specific factors were generally low (see Table 6), indicating that after controlling for the general factor, the reliable specific variance retained by each subscale was limited. The subscales are therefore best used for descriptive or exploratory purposes rather than independent interpretation. For scoring purposes, these findings support prioritizing the total score of the Chinese DSES. Although the specific factors still retain some content-related meaning, the structural evidence suggests that subscale scores should be treated as supplementary descriptions rather than as primary indicators on a par with the total score.

### 3.5. Association with Digital Maturity

To provide additional evidence for the preliminary convergent and external validity of the Chinese version of the DSES, we further examined its association with digital maturity. Theoretically, higher digital self-efficacy should be associated with greater digital maturity; a significant positive correlation was therefore expected.

The correlation analysis showed that the total score of the Chinese version of the DSES was significantly positively correlated with the total score of digital maturity (*r* = 0.642, *p* < 0.001). This indicates that first-year college students with higher digital self-efficacy also tended to report higher digital maturity. By conventional benchmarks, this correlation falls in the moderate-to-large range, indicating a substantial but non-redundant association between the two constructs (Lovakov & Agadullina, 2021). This finding provides preliminary convergent and external validity evidence for the Chinese DSES. Because this evidence is based on a single self-report external correlate collected in the same survey session, it should be interpreted as initial rather than definitive, and future studies should seek to replicate the association using more diverse and methodologically independent criteria (e.g., performance-based digital tasks or informant ratings).

### 3.6. Measurement Invariance Across Gender

Based on the retained Bifactor-ESEM model, measurement invariance across gender was examined using the total sample. Following standard procedures for multi-group measurement invariance testing, configural, metric, and scalar invariance models were tested sequentially across male and female students. The configural invariance model examined whether the same latent factor structure was acceptable in both gender groups. The metric invariance model further constrained factor loadings to be equal across gender groups, and the scalar invariance model additionally constrained item intercepts to equality. Because chi-square difference tests are highly sensitive to large sample sizes, invariance decisions were based primarily on changes in approximate fit indices, including ΔCFI, ΔRMSEA, and ΔSRMR. Following F. F. Chen’s (2007) recommendations, measurement invariance was considered supported when ΔCFI did not exceed 0.010, ΔRMSEA did not exceed 0.015, and ΔSRMR remained within the recommended cutoff values (F. F. Chen, 2007).

As shown in Table 7, the configural invariance model showed good fit to the data, χ^2^(330) = 582.596, CFI = 0.986, TLI = 0.975, RMSEA = 0.032, and SRMR = 0.012, indicating that the Bifactor-ESEM structure was acceptable for both male and female students. When factor loadings were constrained to equality across gender, the metric invariance model also showed good fit, χ^2^(444) = 713.317, CFI = 0.985, TLI = 0.980, RMSEA = 0.028, and SRMR = 0.022. Compared with the configural model, changes in fit indices were negligible, ΔCFI = −0.001, ΔRMSEA = −0.004, and ΔSRMR = 0.010, all of which were within recommended thresholds. These results supported metric invariance across gender.

The scalar invariance model, which further constrained item intercepts to equality, also demonstrated good model fit, χ^2^(463) = 737.173, CFI = 0.985, TLI = 0.980, RMSEA = 0.028, and SRMR = 0.024. Compared with the metric invariance model, changes in model fit were minimal, ΔCFI = 0.000, ΔRMSEA = 0.000, and ΔSRMR = 0.002. These changes did not exceed the recommended cutoff criteria, supporting scalar invariance across gender.

Taken together, the results supported configural, metric, and scalar invariance of the Chinese DSES across gender. These findings suggest that the scale has an equivalent measurement structure across male and female students, that item-factor relationships are comparable, and that item intercepts are sufficiently invariant. Therefore, the Chinese DSES can be considered psychometrically comparable across gender groups in this sample. Importantly, this conclusion concerns measurement equivalence rather than substantive gender differences. The present study did not conduct latent mean comparisons and therefore cannot determine whether male and female students differ in their overall level of digital self-efficacy. Rather, the invariance results establish that such comparisons would be methodologically defensible in future research.

This distinction is important for interpreting the gender-related findings cautiously. The present results should not be read as evidence for or against actual gender differences in digital self-efficacy levels. Instead, they indicate that the Chinese DSES operates similarly across male and female students, providing preliminary support for the fairness and comparability of the scale across gender groups. Future studies may build on this invariance evidence by conducting theoretically guided latent mean analyses, examining whether gender differences vary across educational contexts or disciplines, and incorporating external criteria such as digital task performance, behavioral engagement data, or teacher ratings. Such work would help clarify whether gender is associated with differences in the level or organization of digital self-efficacy beyond measurement equivalence.

## 4. Discussion

### 4.1. Latent Structural Characteristics of the Chinese Version of the DSES

The main finding of this study is that the Chinese DSES was best represented by a Bifactor-ESEM model rather than by a simple five-factor oblique model or a traditional second-order model. Although the exploratory analysis suggested a four-factor solution, this structure was difficult to interpret theoretically because several items from different DigComp domains merged and some items showed cross-domain loadings. In the confirmatory sample, the Bifactor-ESEM model provided the most defensible representation of the data, indicating that item responses were primarily explained by a broad digital self-efficacy dimension, with only modest residual domain-specific components.

This finding clarifies the structural interpretation of the Chinese DSES. The results do not suggest that the five DigComp domains are conceptually irrelevant. Rather, they indicate that, in this sample, these domains function better as content areas for organizing item coverage than as fully independent latent dimensions. Students’ responses appeared to reflect a general belief in their ability to manage digital tasks across contexts more strongly than differentiated confidence in five separate domains.

A key issue is whether the scale should be interpreted as hierarchically organized or as reflecting a strong overarching factor alongside weaker multidimensionality. The present data support the latter interpretation. In a second-order model, the general factor influences items indirectly through first-order domain factors. In contrast, the Bifactor-ESEM model allows the general factor to directly explain item responses while estimating residual domain-specific variance. Because the second-order CFA model fit the data substantially worse than the Bifactor-ESEM model, the general factor appears to be more than a higher-order abstraction of five separable domains. Together with the high omega hierarchical and explained common variance values, the findings provide evidence of a strong overarching digital self-efficacy factor with weaker but still meaningful domain-specific differentiation, rather than treating the construct as strictly unidimensional.

Accordingly, the total score should be treated as the primary unit of interpretation. Subscale scores may still be reported for supplementary or exploratory profiling, but they should not be interpreted as independent indicators equivalent to the total score unless further evidence supports their reliability, stability, and external validity.

### 4.2. Gender Measurement Equivalence: Evidence and Implications

The gender measurement invariance analyses showed that the Chinese DSES demonstrated configural, metric, and scalar invariance across male and female students. The configural model confirmed that the same Bifactor-ESEM factor structure was acceptable in both gender groups. The metric model further showed that item-factor relationships (i.e., factor loadings) were statistically comparable across gender, and the scalar model indicated that item intercepts were sufficiently equivalent. These findings collectively establish that the Chinese DSES produces measurements that are psychometrically comparable across gender groups in this sample.

It is important to interpret these invariance results in terms of what they do and do not establish. The present findings provide measurement equivalence evidence—specifically, that the scale operates through the same latent structure for male and female students, and that meaningful score comparisons between gender groups would be methodologically defensible. However, these results do not constitute evidence for or against substantive gender differences in digital self-efficacy levels. The present study did not conduct latent mean comparisons, and therefore the question of whether male and female first-year students differ in their overall or domain-specific digital self-efficacy remains open.

Furthermore, the finding of scalar invariance should not be overgeneralized to populations beyond the present sample of first-year students from two universities in Guangdong Province. Whether gender measurement equivalence holds across different educational contexts, disciplines, or regional settings would need to be examined in future research. Future studies may build on this invariance evidence by conducting theoretically guided latent mean analyses, by examining whether gender differences—if present—vary across academic disciplines or institutional types, and by incorporating objective indicators such as digital task performance, learning platform behavioral data, or teacher ratings. Such work would help clarify whether gender is associated with differences in the level or organization of digital self-efficacy beyond measurement equivalence.

### 4.3. Possible Reasons for the Emergence of a Strong General Factor

The prominence of the general digital self-efficacy dimension may reflect the integrated nature of students’ digital learning experiences. In contemporary university settings, students often use the same platforms to search for information, communicate with peers, submit assignments, verify identity, and solve technical problems. As a result, they may experience digital tasks as interconnected rather than as clearly separated domains. This may explain why their responses were strongly organized around a general sense of digital confidence.

The developmental stage of first-year college students may also contribute to this pattern. Students entering university are adapting to more self-directed and digitally mediated learning environments. At this stage, they may not yet have developed highly differentiated self-evaluations across specific digital domains. Instead, they may rely on a broader judgment of whether they can manage digital demands in general.

At the same time, this interpretation should remain cautious. Specifically, it is important to acknowledge that the observed structural pattern may not be uniquely attributable to the Chinese educational context. The integration of comprehensive digital platforms is a global trend in higher education, and the strong overarching factor may reflect a broader psychometric characteristic of the DSES across different cultural settings rather than a culturally specific phenomenon. Furthermore, the strong general factor may partly reflect substantive digital self-efficacy, but it may also be strengthened by common method variance because all DSES items used the same self-report format and were collected in a single survey session. Therefore, future studies should examine the general factor using more diverse methods, such as performance-based digital tasks, learning platform data, teacher ratings, or multi-method validation designs.

### 4.4. Value of Bifactor-ESEM for Scale Validation

The findings demonstrate the value of using ESEM and Bifactor-ESEM in validating adapted psychological scales. Traditional CFA may overestimate factor correlations because it forces all non-target loadings to zero. In this study, allowing theoretically plausible cross-loadings through ESEM reduced factor overlap, and adding a general factor through Bifactor-ESEM further clarified the structure of the scale.

Importantly, Bifactor-ESEM contributed not only to better model fit but also to score interpretation. The bifactor reliability indices showed that most reliable variance was attributable to the general digital self-efficacy dimension, whereas the specific factors retained limited reliable variance after controlling for the general factor. Thus, the model results directly support the recommendation that researchers and practitioners prioritize the total score when using the Chinese DSES.

The significant association between the Chinese DSES total score and digital maturity provides preliminary convergent validity evidence. However, this evidence remains limited because both constructs were assessed through concurrent self-report measures. Future studies should use more independent external criteria to further evaluate the validity of the scale.

### 4.5. Limitations and Future Directions

First, the present sample was drawn from two universities in Guangdong Province, an economically developed coastal region of China. Although the sample included students from both urban and rural backgrounds in roughly equal proportions and spanned diverse academic disciplines across two institution types, the regional context may limit generalizability in at least two respects. First, Guangdong Province has relatively advanced digital infrastructure compared with inland and western regions of China, which may influence baseline levels of digital self-efficacy and affect the structural properties of the scale. Second, both universities are located in an urban center (Guangzhou), and even rural-background students in this sample were enrolled in an urban institutional environment at the time of data collection. Future validation work should recruit samples from multiple geographic regions—including inland, rural, and less-resourced institutional contexts—to examine whether the Bifactor-ESEM structure and the psychometric properties reported here replicate across a broader range of Chinese higher education settings. Consequently, greater caution is required when generalizing the educational implications of this study. Given the reliance on a convenience sample restricted to two institutions in Guangzhou, broader educational policies or targeted interventions should not be generalized solely from these preliminary structural findings without further empirical corroboration in more representative samples.

Second, the cross-sectional design did not allow examination of test–retest reliability or longitudinal stability. Future studies should assess whether the Chinese DSES produces stable scores over time and whether digital self-efficacy changes during students’ transition into university.

Third, external validity evidence was preliminary. Although digital maturity was positively associated with the DSES total score, both measures were self-reported and collected concurrently. Future validation work should include objective digital performance tasks, behavioral learning data, teacher ratings, or peer evaluations.

Fourth, although this study compared four-factor, five-factor, second-order, CFA, ESEM, and bifactor models, the stability of the Bifactor-ESEM solution should be examined in additional samples.

Finally, although the adaptation process included translation–back-translation, expert review, and brief open-ended interviews, more systematic cognitive interviewing and pilot testing would further strengthen evidence for cultural and response-process validity.

## 5. Conclusions

This study adapted the Digital Self-Efficacy Scale into Chinese and examined its psychometric properties among Chinese first-year college students. All 25 items showed satisfactory discrimination and homogeneity. Although parallel analysis suggested a four-factor exploratory solution, this structure was difficult to interpret theoretically and did not fully align with the original DigComp-based content framework.

By comparing seven competing models in an independent confirmatory sample, the study found that the Bifactor-ESEM model provided the most defensible representation of the Chinese DSES. The scale was best characterized by a strong overarching digital self-efficacy factor with weaker but still meaningful domain-specific differentiation. Bifactor reliability indices further supported prioritizing the total score, whereas subscale scores should be interpreted cautiously as supplementary descriptive information rather than as independent indicators.

The Chinese DSES total score was positively associated with digital maturity, providing preliminary convergent evidence. However, external validity remains limited because both measures were self-reported and collected concurrently. In addition, configural, metric, and scalar invariance across gender supported the comparability of the scale for male and female students. Overall, the Chinese DSES appears to be a promising instrument with preliminary psychometric support for assessing digital self-efficacy in this population, with the total score recommended as the primary unit of interpretation.

## Figures and Tables

**Table 1:** Descriptive Statistics and Item Analysis Results for the DSES (Subsample 1, *n* = 750).

Item	*M*	*SD*	Skewness	Kurtosis	CR	CITC	Cronbach’s α If Item Deleted
ise1	4.34	0.93	−0.39	0.64	17.15	0.64	0.973
ise2	4.41	0.96	−0.56	0.86	19.24	0.70	0.973
ise3	4.46	0.98	−0.58	0.70	20.47	0.72	0.972
cse1	4.42	0.95	−0.45	0.66	21.26	0.75	0.972
cse2	4.35	1.00	−0.48	0.51	23.18	0.75	0.972
cse3	4.28	1.03	−0.44	0.34	26.51	0.81	0.972
cse4	4.40	0.99	−0.57	0.82	22.61	0.79	0.972
cse5	4.35	0.98	−0.54	0.90	26.20	0.83	0.972
cse6	4.55	0.91	−0.66	1.46	22.31	0.78	0.972
cse7	4.35	1.02	−0.49	0.51	23.02	0.76	0.972
cse8	4.36	0.95	−0.27	0.29	25.62	0.81	0.972
dse1	4.16	1.04	−0.38	0.45	25.87	0.80	0.972
dse2	4.07	1.09	−0.34	0.21	27.94	0.80	0.972
dse3	4.23	1.06	−0.51	0.56	25.38	0.79	0.972
dse4	3.82	1.19	−0.26	−0.21	21.07	0.66	0.973
sse1	4.19	1.04	−0.43	0.32	23.00	0.74	0.972
sse2	4.33	1.01	−0.52	0.70	22.30	0.75	0.972
sse3	4.38	0.99	−0.60	1.01	22.15	0.78	0.972
sse4	4.34	0.99	−0.50	0.91	22.62	0.78	0.972
sse5	4.24	1.02	−0.41	0.51	25.50	0.79	0.972
pse1	4.04	1.09	−0.26	0.06	29.08	0.79	0.972
pse2	4.04	1.12	−0.30	0.04	25.29	0.76	0.972
pse3	4.03	1.09	−0.35	0.22	25.56	0.76	0.972
pse4	3.81	1.19	−0.20	−0.21	25.10	0.72	0.973
pse5	4.27	1.01	−0.52	0.79	22.90	0.76	0.972

***Note.*** *M* = mean; *SD* = standard deviation; CR = critical ratio based on the extreme-group comparison; CITC = corrected item–total correlation. All CR values were significant at *p* < 0.001.

**Table 2:** Parallel analysis for determining the number of factors (Subsample 1, *n* = 750).

Factor	Actual Eigenvalue (EFA)	95th Percentile Random Eigenvalue	Decision
1	14.97	0.40	Retain
2	1.51	0.31	Retain
3	0.57	0.27	Retain
4	0.36	0.23	Retain
5	0.15	0.20	Drop

***Note.*** EFA = exploratory factor analysis. Factors were retained when the actual eigenvalue exceeded the corresponding 95th percentile random eigenvalue. The first four actual eigenvalues exceeded their random-data thresholds, whereas the fifth did not, supporting a four-factor exploratory solution.

**Table 3:** Comparison of Model Fit Indices for Competing Models (Subsample 2, *n* = 752).

Model	χ^2^ (*df*)	RMSEA [90% CI]	CFI	TLI	SRMR	AIC	BIC
4-factor CFA	855.498 (269)	0.054 [0.050, 0.058]	0.932	0.925	0.046	37,787.954	38,162.396
4-factor ESEM	522.337 (206)	0.045 [0.040, 0.050]	0.964	0.947	0.019	37,149.823	37,815.497
Oblique CFA (5-factor)	760.407 (265)	0.050 [0.046, 0.054]	0.943	0.935	0.044	37,492.571	37,885.391
Second-order CFA	910.904 (270)	0.056 [0.052, 0.060]	0.926	0.918	0.054	37,887.113	38,256.932
ESEM (5-factor)	455.657 (185)	0.044 [0.039, 0.049]	0.969	0.949	0.016	36,875.833	37,638.365
Bifactor CFA	743.981 (250)	0.051 [0.047, 0.056]	0.943	0.932	0.047	37,453.008	37,912.149
Bifactor-ESEM	296.023 (165)	0.032 [0.026, 0.038]	0.985	0.973	0.012	36,782.349	37,637.556

***Note.*** CFA = confirmatory factor analysis; ESEM = exploratory structural equation modeling; Bifactor-ESEM = bifactor exploratory structural equation modeling; χ^2^ = chi-square statistic; *df* = degrees of freedom. RMSEA = root mean square error of approximation; CFI = comparative fit index; TLI = Tucker–Lewis index; SRMR = standardized root mean square residual; AIC = Akaike information criterion; BIC = Bayesian information criterion.

**Table 4:** Factor Correlation Matrices for the Five-Factor Oblique CFA and ESEM Models.

Factor	ISE	CSE	DSE	SSE	PSE
Panel A: Five-Factor Oblique CFA Model					
ISE	1				
CSE	0.89	1			
DSE	0.74	0.86	1		
SSE	0.79	0.87	0.87	1	
PSE	0.65	0.74	0.87	0.87	1
Panel B: Five-Factor ESEM Model					
ISE	1				
CSE	0.44	1			
DSE	0.40	0.45	1		
SSE	0.39	0.66	0.62	1	
PSE	0.24	0.53	0.66	0.73	1

***Note.*** ISE = information and data literacy; CSE = communication and collaboration; DSE = digital content creation; SSE = safety; PSE = problem-solving. All correlation coefficients are significant at *p* < 0.001.

**Table 5:** Standardized General and Target-Specific Factor Loadings in the Bifactor-ESEM Model.

Item	Theoretical Domain	General Factor	Target-Specific Factor	Target Loading
ise1	ISE	0.69	ISE	0.41
ise2	ISE	0.74	ISE	0.40
ise3	ISE	0.73	ISE	0.42
cse1	CSE	0.74	CSE	0.39
cse2	CSE	0.70	CSE	0.46
cse3	CSE	0.76	CSE	0.36
cse4	CSE	0.76	CSE	0.32
cse5	CSE	0.82	CSE	0.33
cse6	CSE	0.80	CSE	0.27
cse7	CSE	0.80	CSE	0.04
cse8	CSE	0.83	CSE	0.15
dse1	DSE	0.82	DSE	0.43
dse2	DSE	0.80	DSE	0.36
dse3	DSE	0.80	DSE	0.14
dse4	DSE	0.62	DSE	0.25
sse1	SSE	0.79	SSE	0.32
sse2	SSE	0.79	SSE	0.43
sse3	SSE	0.83	SSE	0.26
sse4	SSE	0.83	SSE	0.02
sse5	SSE	0.82	SSE	−0.03
pse1	PSE	0.81	PSE	0.36
pse2	PSE	0.76	PSE	0.45
pse3	PSE	0.74	PSE	0.48
pse4	PSE	0.65	PSE	0.57
pse5	PSE	0.72	PSE	0.27

***Note.*** ISE = information and data literacy; CSE = communication and collaboration; DSE = digital content creation; SSE = safety; PSE = problem-solving. The general factor represents overall digital self-efficacy. Target-specific factor loadings are the loadings of each item on its theoretically assigned domain-specific factor after controlling for the general factor. The full standardized loading matrices for the five-factor ESEM and Bifactor-ESEM models, including all non-target cross-loadings, are provided in Appendix A.

**Table 6:** Hierarchical Reliability and Bifactor Dimensionality Indices for the Bifactor ESEM.

Score/Factor	ω	ω_H_/ω_HS_	ECV
Total score	0.983	0.946	—
ISE	0.885	0.235	0.035
CSE	0.953	0.121	0.053
DSE	0.911	0.130	0.027
SSE	0.934	0.057	0.024
PSE	0.936	0.247	0.065
G	—	—	0.797
PUC (conventional count based on theoretical item-domain assignment)			0.810

***Note:*** ω = omega total; in the “Total score” row, the hierarchical omega corresponds to ω_H_, representing the hierarchical omega of the total score; ω_HS_ = hierarchical omega subscale after controlling for the general factor; ECV = explained common variance; PUC = percentage of uncontaminated correlations. The PUC value was computed using the conventional item-domain assignment and is reported as a supplementary descriptive index; it was not used as the primary basis for interpretation in the Bifactor-ESEM framework. The computation and interpretation of these indices followed Rodriguez et al. (2016).

**Table 7:** Gender Measurement Invariance of the Bifactor-ESEM Model.

Model	χ^2^	*df*	CFI	TLI	RMSEA	SRMR	ΔCFI	ΔRMSEA	ΔSRMR
Configural invariance	582.596	330	0.986	0.975	0.032	0.012	—	—	—
Metric invariance	713.317	444	0.985	0.980	0.028	0.022	−0.001	−0.004	0.01
Scalar invariance	737.173	463	0.985	0.980	0.028	0.024	0	0	0.002

***Note.*** CFI = comparative fit index; TLI = Tucker–Lewis index; RMSEA = root mean square error of approximation; SRMR = standardized root mean square residual. Δ values indicate changes relative to the preceding invariance model. Following F. F. Chen (2007), invariance was evaluated primarily using changes in approximate fit indices rather than chi-square difference tests.

## Data Availability

The authors had full control of all the primary data and the datasets used and analyzed during the current study are available from the corresponding author on reasonable request.

## Appendix A

**Table A1:** Rotated Factor Loading Matrix for the Four-Factor Exploratory Factor Analysis (EFA) for Subsample 1.

Item	Factor 1	Factor 2	Factor 3	Factor 4
ISE1	0.753 *	−0.009	−0.027	0.026
ISE2	0.717 *	−0.063	0.180 *	−0.029
ISE3	0.800 *	−0.009	0.050	−0.002
CSE1	0.859 *	0.020	−0.030	0.029
CSE2	0.763 *	−0.005	−0.048	0.176 *
CSE3	0.699 *	0.125	−0.039	0.169 *
CSE4	0.720 *	0.043	0.075	0.087
CSE5	0.643 *	0.155 *	0.016	0.168 *
CSE6	0.570 *	0.196 *	0.278 *	−0.134 *
CSE7	0.327 *	0.338 *	0.326 *	−0.092 *
CSE8	0.422 *	0.405 *	0.139	−0.014
DSE1	0.087	0.789 *	−0.008	0.094
DSE2	0.031	0.778 *	−0.018	0.182
DSE3	0.138 *	0.440 *	0.278 *	0.088
DSE4	−0.060	0.388 *	0.048	0.438 *
SSE1	−0.052	0.251 *	0.624 *	0.075
SSE2	−0.013	0.083	0.775 *	0.063
SSE3	0.131	−0.015	0.789 *	0.039
SSE4	0.208	−0.025	0.544 *	0.213 *
SSE5	0.197	−0.004	0.434 *	0.326 *
PSE1	0.010	0.104	0.263 *	0.590 *
PSE2	−0.017	0.074	0.147 *	0.745 *
PSE3	0.092	−0.005	0.044	0.823 *
PSE4	0.029	0.037	−0.034	0.877 *
PSE5	0.355 *	−0.006	0.064	0.501 *

***Note.*** *N* = 750. MLR estimation and Geomin (oblique) rotation were used. Factor loadings with absolute values ≥ 0.30 are shown in bold to highlight primary loadings and significant cross-loadings. * *p* < 0.05.

**Table A2:** Standardized Loading Matrices for the Five-Factor ESEM and Bifactor-ESEM Models.

Item	ESEM					G	Bifactor-ESEM				
	ISE	CSE	DSE	SSE	PSE		ISE	CSE	DSE	SSE	PSE
**ise1**	0.33	0.48	−0.10	0.07	0.18	**0.69**	**0.41**	0.15	−0.03	−0.07	−0.08
**ise2**	0.34	0.33	−0.11	**0.31**	0.13	**0.74**	**0.40**	0.09	−0.05	0.08	−0.07
**ise3**	0.42	0.44	−0.08	0.10	0.15	**0.73**	**0.42**	0.23	−0.03	0.00	−0.06
**cse1**	0.47	0.46	0.05	0.07	0.07	**0.74**	**0.28**	**0.39**	−0.02	0.05	−0.06
**cse2**	**0.38**	0.43	0.19	−0.05	0.11	**0.70**	0.13	**0.46**	0.03	0.02	0.02
**cse3**	**0.27**	**0.43**	**0.25**	0.05	0.08	**0.76**	0.08	**0.36**	0.09	0.02	0.00
**cse4**	0.17	**0.48**	**0.26**	0.12	−0.01	**0.76**	0.02	**0.32**	0.03	0.00	−0.07
**cse5**	0.13	**0.50**	**0.26**	0.14	0.03	**0.82**	−0.05	**0.33**	0.02	−0.02	−0.06
**cse6**	0.07	**0.67**	0.10	0.13	0.01	**0.80**	0.00	0.27	−0.11	−0.14	−0.16
**cse7**	−0.01	0.39	0.18	**0.41**	−0.04	**0.80**	0.03	0.04	0.03	0.05	−0.11
**cse8**	0.03	**0.50**	0.23	0.18	0.06	**0.83**	0.02	0.15	0.07	−0.05	−0.07
**dse1**	−0.09	0.41	**0.55**	−0.01	0.14	**0.82**	0.01	0.03	**0.43**	−0.10	0.01
**dse2**	−0.10	0.32	**0.61**	−0.01	0.18	**0.80**	−0.06	0.06	**0.36**	−0.05	0.12
**dse3**	−0.08	0.25	0.35	**0.33**	0.08	**0.80**	−0.05	0.01	0.14	0.06	0.04
**dse4**	0.03	−0.15	**0.43**	0.12	**0.39**	**0.62**	−0.08	0.00	**0.25**	0.13	**0.35**
**sse1**	0.11	−0.07	**0.25**	**0.66**	0.02	**0.79**	−0.04	0.03	0.06	**0.32**	0.08
**sse2**	0.18	−0.14	0.07	**0.89**	−0.05	**0.79**	0.04	−0.02	−0.02	**0.43**	0.02
**sse3**	0.04	0.08	−0.02	**0.79**	0.04	**0.83**	−0.01	−0.06	−0.11	0.26	−0.02
**sse4**	−0.09	**0.31**	0.01	**0.44**	**0.23**	**0.83**	−0.09	−0.04	−0.10	0.02	0.04
**sse5**	−0.14	**0.24**	−0.10	**0.40**	**0.47**	**0.82**	−0.03	**−0.19**	−0.08	−0.03	**0.14**
**pse1**	0.02	0.01	0.04	**0.24**	**0.66**	**0.81**	−0.03	−0.09	0.01	0.04	**0.36**
**pse2**	0.02	−0.02	0.04	0.10	**0.79**	**0.76**	−0.01	−0.11	0.02	−0.02	**0.45**
**pse3**	0.01	−0.06	**0.12**	0.06	**0.80**	**0.74**	−0.06	−0.10	0.08	−0.02	**0.48**
**pse4**	0.10	**−0.19**	**0.26**	−0.06	**0.80**	**0.65**	−0.07	−0.01	**0.17**	0.04	**0.57**
**pse5**	0.11	0.13	0.13	0.12	**0.45**	**0.72**	−0.03	0.10	−0.01	0.02	0.27

***Note.*** ISE = information and data literacy specific factor; CSE = communication and collaboration specific factor; DSE = digital content creation specific factor; SSE = safety specific factor; PSE = problem-solving specific factor; G = general factor. All loadings are standardized. Boldface indicates statistically significant loadings (* *p* < 0.05).

## Appendix B

The Chinese version of the Digital Self-Efficacy Scale (DSES) items listed in this appendix was translated and revised by the research team based on the original English version of the scale (Ulfert-Blank & Schmidt, 2022). The scale consists of five competence domains: (1) 信息和数据素养 (ISE); (2) 沟通和协作 (CSE); (3) 数字内容创作 (DSE); (4) 安全 (SSE); (5) 解决问题 (PSE)。

1. Responses to the scale items are rated using a six-point Likert scale, as shown below:
  (1) 完全不同意
  (2) 不同意
  (3) 稍微不同意
  (4) 稍微同意
  (5) 同意
  (6) 完全同意
2. Chinese Version of the DSES Items:
  ISE1: 我自信能够在数字环境中搜索特定信息。
  ISE2: 我自信能够区分正确的和错误的数字信息。
  ISE3: 我自信能够存储并整理数字内容，以便我能轻松地再次找到它们。
  CSE1: 我自信能够在数字环境中与他人互动。
  CSE2: 我自信能够以数字方式与他人分享信息和数据。
  CSE3: 我自信能够参与数字环境中的公共讨论和活动。
  CSE4: 我自信我能在数字环境中维护自身免受不公正对待。
  CSE5: 我自信能够使用数字系统与他人协作。
  CSE6: 我自信能够在数字环境中使用得体的礼仪进行沟通。
  CSE7: 我自信能够管理和删除我的数字足迹。
  CSE8: 我自信能够在数字环境中以我想要的方式展示自己。
  DSE1: 我自信能够创建数字内容。
  DSE2: 我自信能够修改数字内容以生成新的内容。
  DSE3: 我自信能够识别数字环境中的法律相关问题，如使用条款和许可。
  DSE4: 我自信能够用编程语言编写一条简单的命令。
  SSE1: 我自信我能保护我的数字设备免受未经授权的访问。
  SSE2: 我自信我能保护我在数字环境中的个人数据。
  SSE3: 我自信能够认识到使用数字环境可能带来的健康风险。
  SSE4: 我自信我能利用数字环境促进我的健康。
  SSE5: 我自信能够认识到数字环境对自然和气候的影响。
  PSE1: 我自信能够识别使用数字环境时遇到的技术问题。
  PSE2: 我自信能够找到并应用多种解决方案来应对出现的技术问题。
  PSE3: 我自信能够找到合适的数字系统来应对非技术性挑战。
  PSE4: 我自信能够开发新颖的数字解决方案。
  PSE5: 我自信能够识别并提升我所缺乏的数字技能。
